# Topological data analysis of high resolution diabetic retinopathy images

**DOI:** 10.1371/journal.pone.0217413

**Published:** 2019-05-24

**Authors:** Kathryn Garside, Robin Henderson, Irina Makarenko, Cristina Masoller

**Affiliations:** 1 School of Mathematics, Statistics and Physics, Newcastle University, Newcastle upon Tyne, United Kingdom; 2 Department of Physics, Universitat Politecnica de Catalunya, Barcelona, Spain; Normandie Universite, FRANCE

## Abstract

Diabetic retinopathy is a complication of diabetes that produces changes in the blood vessel structure in the retina, which can cause severe vision problems and even blindness. In this paper, we demonstrate that by identifying topological features in very high resolution retinal images, we can construct a classifier that discriminates between healthy patients and those with diabetic retinopathy using summary statistics of these features. Topological data analysis identifies the features as connected components and holes in the images and describes the extent to which they persist across the image. These features are encoded in persistence diagrams, summaries of which can be used to discrimate between diabetic and healthy patients. The method has the potential to be an effective automated screening tool, with high sensitivity and specificity.

## Introduction

The world population is aging, which carries a high risk of eye diseases that significantly affect the quality of life. Thus, many efforts are nowadays focused on the development of reliable data analysis tools able to improve early diagnosis and follow-up of eye diseases. Diabetic retinopathy (DR) is a particularly relevant disease. In 2013, 382 million people had diabetes and this number is expected to rise to 592 million by 2035 [1]. DR is a complication of diabetes and the most frequent cause of new cases of blindness in developed countries among adults aged 20–74 years [2]. Timely identification is crucial for the success of the treatment. DR identification is based on the inspection of the fundus color image, which in the early stage of the disease has no visible symptoms, but as DR advances, the structure of the blood vessels changes and microaneurysms, exudates and new blood vessels can be seen. Depending on the population size among other factors, remote screening for the detection of the disease is not always cost-effective [3], partially due to the cost of the eye physicians that remotely analyse the images [4].

In recent years a wide variety of automated methods for the detection of the disease have been proposed in the literature [5–12] but their success varies significantly with the retinal image modality, image quality and the number of images available. Here we use a small publicly available high resolution fundus image database [13] to demonstrate that new techniques based on recent developments in topological data analysis (TDA) can be used to extract informative features from the images and lead to effective classification.

### Topological data analysis

Topological data analysis considers the shape of data and its topological properties. At the intersection of algebraic topology and computational geometry, TDA provides methods to describe data using low dimensional topological invariants. One emergent branch of this field is persistent homology, which considers how features of the data persist at different scales.

Homology is the association of algebraic objects, such as abelian groups, to other mathematical objects, such as topological spaces. The homology groups of a space represent the n-dimensional holes in that space. Persistent homology is the multiscale perspective of these homology groups when applied to data. Connectedness is characterised by the 0-th homology group *H*_0_, loops (1-dimensional holes), are given by *H*_1_ and voids (2-dimensional holes) by *H*_2_. There are higher dimensional analogues of the homology groups but their interpretation is not as intuitive. The ranks of these homology groups are also known as the Betti numbers (*β*_*n*_). The maximum dimension of these features is limited by the dimension of the data. For *n*-dimensional data, the maximum dimension of the simplicies is also *n*, which only allows for (*n* − 1)-dimensional features to be observed. In our analysis of two-dimensional images we only consider 0- and 1-dimensional features, that is components and holes.

A representation of the data is constructed from simplices (vertices, edges, triangles, tetrahedra etc.) according to the sub-level sets of some filtration function. During its construction we observe changes in the homology and describe these changes in terms of their *birth* and *death*. The lifetime of these features is presented as either a persistence diagram or barcode.

#### Illustration

**Fig 1:**
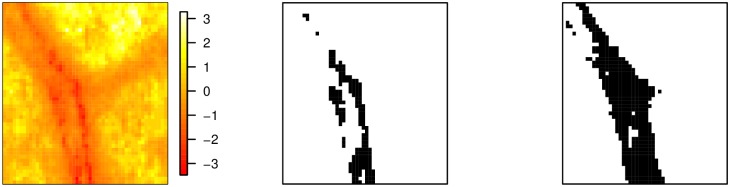
Example 50 × 50 field (left) with level sets corresponding to *ℓ* = −1.5 (centre) and *ℓ* = −0.9 (right).

We present a simple example to illustrate how persistent homology is applied to an image. A 50 × 50 subset of one of the fundus images is presented as a 2D field in the left part of Fig 1. Let *z*(*x*, *y*) denote the field value at location (*x*, *y*). In the example, the values of *z*(*x*, *y*) range from -3.37 to 3.17 and represent the grayscale encoding of the image.

We consider a filtration constructed by increasing a level *ℓ* from below the smallest value in the field to above the largest value. The sub-level set associated with any value of *ℓ* consists of all locations (*x*, *y*) for which *z*(*x*, *y*) < *ℓ*. The central and right panels of Fig 1 show, in red, the sub-level sets corresponding to *ℓ* = −1.5 and *ℓ* = −0.9 respectively. In the central panel there are five groups of connected components but no holes, so the first two Betti numbers are *β*_0_ = 5 and *β*_1_ = 0. By level *ℓ* = −0.9 the components in the previous sub-level set have merged with each other and with other locations to form one large component, but four new and smaller components have been created. Hence in this plot *β*_0_ = 5. The main component now also contains three holes (or islands) and *β*_1_ = 3.

**Fig 2:**
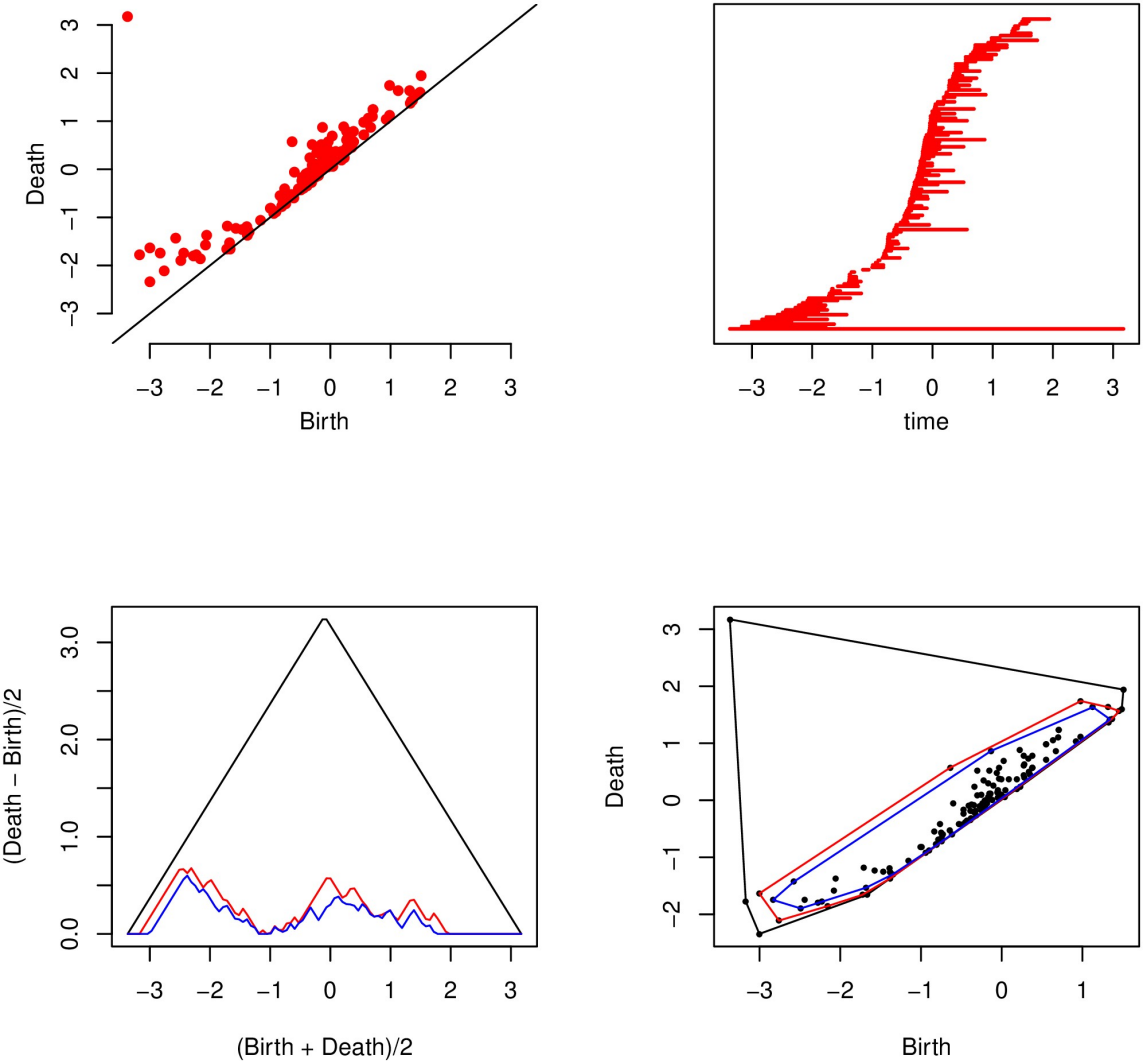
Dimension 0 persistence diagram (top left), persistence barcode (top right), first three landscape functions (bottom left) and first three convex peels (bottom right) for the data in Fig 1.

As level *ℓ* increases, components and holes are born and die. For fields in two dimensions, a new component is born at a local minimum of the field *z*(*x*, *y*) and a hole dies at a local maximum. The standard labelling convention when features merge is to assume the feature with the earliest birth time continues and the feature with the later birth time dies. A persistence diagram is then a plot of birth time against death time for features of a particular type. The top left panel of Fig 2 illustrates with a persistence diagram for components created during a filtration of the field in Fig 1. The first component to be born is by construction the last to die: this is the point in the top-left of the plot. Points close to the diagonal represent features with a short lifespan, whilst points furthest from the diagonal represent longlived features. It is tempting to dismiss the short-lived features as uninformative noise in the data, but these points represent the local inhomegeneity. In applications such as this, these points can provide important information. The high resolution of the data provides access to this level of fidelity.

A persistence barcode is also presented in the top right panel of Fig 2. The lifetime of a feature is encoded in the length of each bar, which extends from the birth to death time of a feature. There is a single bar that extends the full length of the barcode representing the first component to be born, which is also the last to die.

A summary of the basic shape of a persistence diagram using convex peels has been proposed [14]. Convex hulls of the point cloud are successively peeled away until only a specified proportion remains. The bottom right panel of Fig 2 illustrates this, showing a convex hull around 90% of the points, together with the two peeled convex hulls. This summary aims to extract the overall shape of the points in the persistence diagram without undue influence of either outliers, or the density of points along the diagonal.

An alternative function known as the landscape has also been proposed [15] and is illustrated in the bottom left panel of Fig 2. The points in a persistence diagram are first rotated from birth death pairs (*b*, *d*) to (*x*, *y*) = ((*d* + *b*)/2, (*d* − *b*)/2). A piecewise linear tent function is then created for each point and the *k*th landscape function at any point *t* on the horizontal axis is the *k*th largest value of the tents. The landscape function is able to distinguish the two apparent clusters in the diagram, but it is more sensitive to small variations in persistent points than is the convex peel.

Another proposed functional summary of the persistence diagram is the accumulative persistence function [16]. The persistence diagram is now rotated about the diagonal and rescaled such that birth death pairs (*b*, *d*) are transformed to (*x*, *y*) = ((*d* + *b*)/2, *d* − *b*). The accumulative persistence function is a one dimensional function calculated as the integral, or accumulative sum, of this transformed diagram. A relatively smooth function indicates that there are similar levels of features at all scales in the data, whilst a distinct jump in the function indicates a disparity between the scales, for example a distinct and large feature is present relative to low levels of background noise. An illustration of this function is not given however, as this descriptor was not selected for the final DR classifier.

The bottleneck and Wasserstein distances are popular methods of distinguishing persistence diagrams, defined respectively as a form of maximum and average distance between points in an optimal bijective mapping between two diagrams. These metrics can be useful for pairwise comparison, albeit at large computational cost when the diagrams are large. Due to the large number of points in the persistence diagrams in this study, computing the inter and intra group bottleneck or Wasserstein distributions is computationally infeasible. The computation of a single bottleneck distance between components of two persistence diagrams in this study was completed in 128 minutes on a high performance computer with 128 GB memory allocation. Complete pairwise comparision of all diagrams in this study would require $2\times \left(\begin{array}{c} 30\\ 2 \end{array}\right)=870$ calculations. As such, we consider functional and interpretable statistics on individual persistence diagrams rather than pairwise comparisons. Our aim is to use these as classification and discrimination tools for the allocation of images to healthy or diabetic groups.

## Materials and methods

### Retinal fundus images

**Fig 3:**
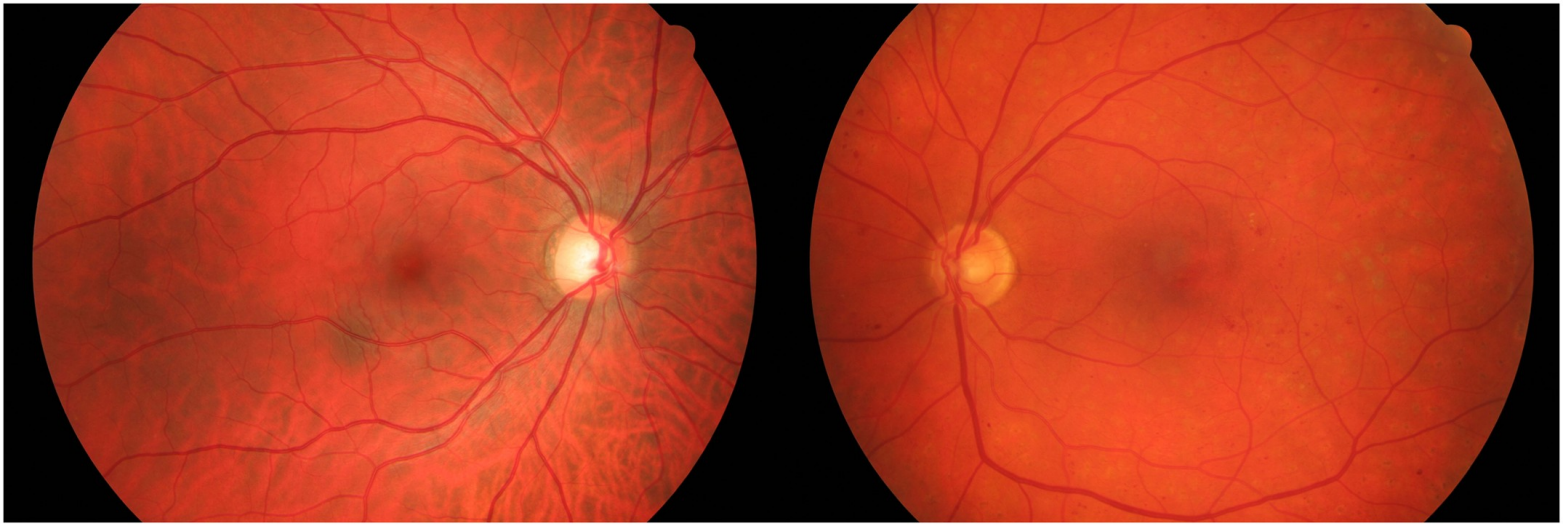
Fundus images from specimen healthy (left) and diabetic retinopathy (right) patients. These are low resultion versions of high resolution images available from the website https://www5.cs.fau.de/research/data/fundus-images/ associated with [13].

Images were obtained from the High-Resolution Fundus (HRF) Image Database [13], which was established in order to support studies on automatic vessel segmentation algorithms. The database includes, inter alia, high resolution fundus images from 15 healthy patients and 15 patients with diabetic retinopathy. Low resolution versions of two of the images are given in Fig 3. We used low resolution images for presentation here simply to keep the file sizes manageable: the original images are each around 2Mb in size with resolution 2336 × 2604. We used all 30 full resolution images in the analyses to follow.

### Pre-processing and persistent homology

The images were first cropped in order to minimise the surrounding border and converted to greyscale. Each image was then marginally transformed to a normal distribution and standardised before computing the persistent homology. This process allowed an alignment of the images such that they could be compared within the same frame of reference. The transformed images were identical in the marginal properties of mean, range and distribution of field values.

Persistence diagrams for each image were computed using the lower star filtration method within the Dionysus 2 software package http://www.mrzv.org/software/dionysus2/. This method triangulates a grid of values according to the sub-level sets of the function values on the grid. In the retinal fundus images this function is the greyscale encoding of the image, which was computed using a linear combination of the red, green and blue components in the image with the coefficients 0.299, 0.587 and 0.114, respectively. The lower star filtration method is an appropriate approach for image or gridded data as it filters the image based on the function value at each pixel. An alternative upper star filtration method refers to the super-level sets of the function value at each pixel. These methods are symmetric with respect to the function valued grid.

### Summary statistics

We summarised persistence diagrams for both components and holes through vectors made up of the following interpretable statistics.

1. The number of points.
2. The average lifetime (death time − birth time).
3. Area under the accumulative persistence function.
4. For the six polygons determined by each of the first three landscape functions and for 99%, 95% and 90% convex peels:
  - the centroids, *C*_*x*_ and *C*_*y*_;
  - the area *A*;
  - the perimeter *P*;
  - the filamentarity
    $$\begin{array}{c} F=\frac{P^{2}-4\pi A}{P^{2}+4\pi A}. \end{array}$$

This gave vectors of length 2 × (1 + 1 + 1 + 6 × 5) = 66.

### Classification

A support vector machine (SVM) was used to classify the images according to the persistence function summary statistics. A least absolute shrinkage and selection operator method (LASSO) was used to perform variable selection for the SVM. LASSO is a method of regression analysis that can be used to perform variable selection as it enforces a penalty term such that certain coefficients are set to zero. This allowed optimal feature selection from the feature vector of length 66 such that the dimension of the SVM was as low as possible, whilst retaining good power of discrimination between the two groups. To assess the performance of the SVM we performed cross validation by splitting the data into a training set, on which the classifier would be trained, and a test set on which the SVM makes a prediction. We calculated sensitivity and specificity as the rate of correct identification of diabetic and healthy subjects respectively. The type of cross validation we performed is called leave-k-out cross validation. We used randomly chosen test sets with the number of subjects equal to 1, 2, 3, 4 & 5 and repeatedly trained the SVM on the remaining data, testing the predictions across all permutations of the random training and test sets.

All persistence diagram summary statistics that we have presented, as well as the SVM, can be computed using publically available packages in R.

## Results

Variables for two of the optimal models identified via the LASSO method are presented in Table 1 and the subsequent sensitivity and specificity results are given in Table 2. The two LASSO-informed models were selected based on the minimum error and where the error is within one standard error of this minimum. Other SVM models were tested using alternative combinations of the summary statistics, for example only using landscape or convex peel statistics. However they did not match the LASSO-informed models in terms of sensitivity and specificity. For the two LASSO-informed models, perfect classification was achieved for leave-one-out cross validation, but we note that this is a small dataset and there is a risk of over-fitting. Therefore in order to provide confidence in the methods and results we performed further cross validations, using test sets of sizes 2, 3, 4 & 5. Sensitivity and specificity were reduced with the size of the training set but still a high level of accuracy was maintained.

**Table 1:** Variables included in LASSO-informed SVM.

	SVM 1	SVM 2
Number of Components	x	x
Components 90% Convex Peel *C*_*x*_	x	x
Components 90% Convex Peel P	x	
Components 3rd Landscape A		x
Components 3rd Landscape F	x	
Components Accumlative Persistence A	x	x
Holes 99% Convex Peel *C*_*y*_	x	x
Holes 1st Landscape P	x	x
Holes 2nd Landscape *C*_*y*_	x	
Holes 3rd Landscape *C*_*y*_	x	

**Table 2:** Sensitivity and specificity results for cross validation of LASSO informed models.

Test Set Size	SVM 1		SVM 2	
	Sensitivity	Specificity	Sensitivity	Specificity
1	1.000	1.000	1.000	1.000
2	1.000	0.996	1.000	1.000
3	0.998	0.987	0.998	0.998
4	0.995	0.983	0.995	0.995
5	0.993	0.980	0.991	0.993

Mean and standard deviation for the variables in the LASSO-informed models are presented in Table 3 for diabetic and healthy subjects. The most obvious differences between the groups are in the numbers and area variables for components, with smaller relative differences elsewhere. In most cases we have smaller values for the images of eyes from healthy people. However, there is considerable variability within each group. For example, one of the healthy images had 306782 components and one diabetic image had 169290 components. There is also overlap between the two groups for the other persistence diagram statistics and hence there is no single discriminatory statistic, instead the statistics collectively need to be used in the support vector machine.

**Table 3:** Mean and standard deviation for variables included in LASSO-informed SVM.

	Healthy (Mean ± SD)	Diabetic (Mean ± SD)
Number of Components	207400 ± 50692	312000 ± 80721
Components 90% Convex Peel *C*_*x*_	0.626 ± 0.073	0.696 ± 0.059
Components 90% Convex Peel P	8.569 ± 0.174	8.809 ± 0.241
Components 3rd Landscape A	0.694 ± 0.135	1.049 ± 0.246
Components 3rd Landscape F	0.687 ± 0.051	0.589 ± 0.077
Components Accumlative Persistence A	40030 ± 7252	76172 ± 29296
Holes 99% Convex Peel *C*_*y*_	1.227 ± 0.074	1.397 ± 0.072
Holes 1st Landscape P	8.269 ± 0.243	8.468 ± 0.073
Holes 2nd Landscape *C*_*y*_	0.241 ± 0.043	0.355 ± 0.107
Holes 3rd Landscape *C*_*y*_	0.231 ± 0.046	0.336 ± 0.097

**Fig 4:**
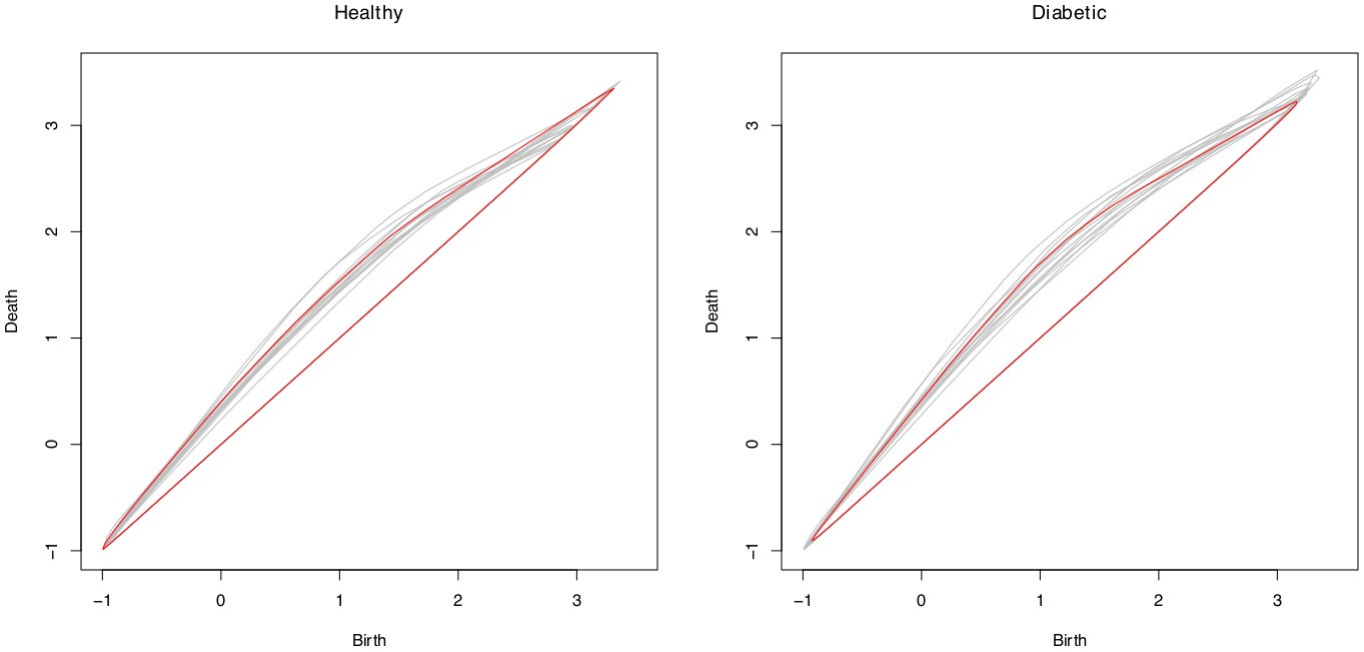
Holes 99% convex peels for healthy (left) and diabetic (right) images. Highlighted in red are the 99% convex peels for the example images in Fig 3.

An example of the difference in persistence diagram statistics between the two groups is presented in Fig 4. A comparison of the 99% convex peels for the holes in the persistence diagrams for healthy and diabetic images is given. The convex peels for the two images in Fig 3 are highlighted in red. We can see a small, but systematic, shift in the centroid of the convex peel between the two groups, which explains why it was selected as a contributing variable in the SVM classifier.

## Discussion and conclusion

We propose an application of supervised SVM learning using summary statistics of retinal fundus image persistence diagrams as an effective method of diabetic retinopathy detection. We recommend, however, that these methods be explored further once larger datasets of high-resolution images become available.

A combination of summary statistics was required in order to provide good classification, with no single persistence diagram summary statistic able to separate entirely the diabetic and healthy images. The vector of summary statistics used in our SVM are decribed in Table 1. We do not include the coefficients of this vector since our method of LOOCV re-trains the SVM with each subject left out and the coefficients of this vector change at each re-training step. Instead we present our results as demonstrative of the potential of TDA for detection of diabetic retinopathy rather than definitive.

The LASSO-informed SVM models, which achieved excellent sensitivity and specificity, suggest that the centroid coordinates of the persistence diagrams are important in classification. A change in the centroid *x*-coordinate means a difference in the average feature birth time, and a change in the *y*-coordinate, given the *x*-coordinate, means a change in the lifetime of features. There is, however, no real difference in the centroid of the original persistence diagram point cloud. Instead, the differences in centroids emerge when we begin removing points from the diagram, for example in the convex peel and landscape functions. The diabetic subjects have higher *x*- and *y*-coordinates for both the convex peel and landscape functions, suggesting that the diabetic subjects may have more extremal points that cause a shift in the centroid as we peel away such points.

The number of components in the persistence diagram also appears to be an informative summary statistic, with the diabetic subjects, on average, having a greater number of connected components than healthy subjects. There is, however, variability within both diabetic and healthy patients such that we cannot use the number of connected components as a single discriminatory statistic. High numbers of (short-lived) components are associated with low local correlation, suggesting the diabetic images are less homogeneous.

Soomro et al [12] presented an excellent survey of DR classifiers and databases, detailing sensitivity, specificity and accuracy results for the respective classifiers and data sources. Our results are at worst comparable and often better than those quoted. Of the main publicly available DR databases, DRIVE [17], STARE [18], ImageRet [19], ROC [20], EyePacs [21] and MESSIDOR [22], there are no data of comparable resolution to the data used in this study. The highest resolution of these data sources is less than half of the resolution of the HRF data (2336 × 2604).

For example, the MESSIDOR data has a resolution of 1440 × 960, resulting in persistence diagrams with approximately 70, 000 components. In comparison the HRF diabetic images have an average of approximately 300, 000 components in their persistence diagrams, and healthy 200, 000. Although this lower resolution makes pairwise comparisons using bottleneck or Wasserstein distances more practical, we found that we were unable to discriminate between healthy and diabetic with high sensitivity and specificity using either these statistics or the SVM classifier developed on the HRF data.

Current DR classification methods can be summarised by three categories: full image, abnormality and segmented retinal blood vessel based detection. Abnormality based detection methods are based on the identification of microaneuryms, haemorrhages and exudates. It is often stated, however, that retinal vascularity is highly indicative of disease development. As such, there is a great deal of work on developing accurate segmentation methods to extract the retinal vessel structures. This study has focussed on full image based DR classification but further work would investigate the power of TDA to detect DR based on vessel structures, utilising larger open source vessel segmentation databases.

To summarize, we have demonstrated that topological data analysis has the potential to become a good discriminator for DR in high resolution fundus images. Further testing using larger numbers of images is of course necessary. At the moment, however, there are no public libraries of very high resolution images other than those described by [13]. Once these become available the combination of TDA and very high resolution images has the potential to be an extremely effective and cost-efficient diagnostic screening tool. In the meantime, we hope to explore how to exploit these methods for lower resolution images. Further work on segmented vessel structures will aim to highlight any difference in DR detection between topological and more traditional methods of data analysis.
